# Ninety Days of Preoperative Endocrine Therapy Informs Patient and Physician Preference for Radiation Therapy: Primary Results from the Preoperative Window of Endocrine Therapy to Inform Radiation Therapy Decisions (POWER) Trial

**DOI:** 10.1245/s10434-025-17861-1

**Published:** 2025-08-02

**Authors:** Trish Millard, Lena M. Turkheimer, Gina Petroni, David Brighton, Max O. Meneveau, Prisca Obidike, Kandace McGuire, Shayna L. Showalter

**Affiliations:** 1 Present Address: Department of Medicine, University of Virginia School of Medicine, Charlottesville, VA USA; 2 Present Address: Department of Surgery, University of Virginia School of Medicine, Charlottesville, VA USA; 3 Division of Translational Research and Applied Statistics, Department of Public Health Sciences, University of Virginia Health System, Charlottesville, VA USA; 4 Department of Surgery, Virginia Commonwealth University Health, Richmond, VA USA

## Abstract

**Background:**

Clinical trial data support the omission of radiation therapy (RT) in older women with early-stage breast cancer. The majority of older women still receive RT largely due to a lack of insight regarding endocrine therapy (ET) tolerance. The POWER trial is a phase II trial designed to determine whether 90 days of preoperative ET (pre-ET) changes patient or surgeon preferences for RT.

**Methods:**

Between 2020 and 2024, two centers enrolled women aged 65 years or older with invasive carcinoma measuring ≤2 cm, N0, and ER+/PR±/HER2−. All participants were recommended for 90 days of pre-ET. Patient and surgeon preferences for RT were evaluated before and after pre-ET. A change in preference was tested from an assumed low change rate of 5% to a rate of ≥15%, with a one-sided 5% level binomial test.

**Results:**

Seventy-five women enrolled. Adverse events attributed to pre-ET occurred in 35 participants (47%). After pre-ET, 21 (28.0%) patients changed RT preference and surgeons changed RT recommendation for 18 (24.0%) patients (*p* < 0.001 and *p* = 0.015, respectively). General agreement between patient and surgeon preferences before pre-ET was 53% (Kappa = −0.04) and increased to 81% (Kappa = 0.59) after pre-ET.

**Conclusion:**

Pre-ET significantly changed patient and surgeon preferences for RT and increased patient and surgeon agreement. The intentional resequencing of treatments enables patients to assess tolerance to ET before deciding whether to omit RT. This study validates pre-ET as an innovative method to inform adjuvant therapy decisions and recommendations.

There are over 125,000 new breast cancer diagnoses among women in the United States each year, and more than 45% of these patients are aged 65 years and older.^1^ For more than 2 decades, breast-conserving surgery (BCS) followed by adjuvant endocrine therapy (AET) has been a widely recommended and commonly utilized treatment approach for many patients with early-stage, estrogen receptor-positive (ER+) breast cancer. Adjuvant radiation therapy (RT) reduces ipsilateral breast tumor recurrences if administered within 8 weeks of BCS.^2–4^ AET is routinely initiated after RT to reduce local recurrence, distant recurrence, and mortality.^5^

As breast cancer treatment has become more personalized, clinical trials have sought to de-escalate treatment for lower-risk patients. Both the CALGB 9343 and PRIME II trials demonstrated that RT could be safely omitted in select patients with ER+ breast cancer for whom at least 5 years of AET was planned. These prospective, randomized, controlled trials enrolled patients (≥70 and ≥65 years of age, respectively) with stage I–II ER+ breast cancer (tumor ≤2 cm and ≤3 cm, respectively) and randomized them to treatment with AET, with or without RT. In both trials, RT omission resulted in no difference in the frequency of mastectomy for recurrence, time to distant metastasis, breast cancer-specific survival, and overall survival.^6–8^ Based on these results, the National Comprehensive Cancer Network guidelines were updated to include the option for RT omission for patients with ER+, human epidermal growth factor receptor 2 (HER2)-negative, node-negative breast cancer with tumors ≤3 cm who were aged ≥65 years.^9^

Despite high-quality evidence supporting RT omission among older women with early-stage ER+ breast cancer, most women in this population continue to be treated with RT.^10–13^ There is growing agreement that RT is not necessary and should be considered ‘overtreatment’.^6,14^ Underlying the hesitancy toward omitting RT is the fact that the rate of non-adherence to AET is 23–28% in the clinical trial setting, and even higher in studies using real-world data.^15–17^ As a result, adjuvant RT is often recommended to mitigate the risk of ‘undertreatment’ in patients who forgo RT and are non-adherent to AET, thereby helping to preserve acceptable oncologic outcomes.^18,19^ Non-adherence to AET is often driven by adverse effects and impaired quality of life, with musculoskeletal symptoms being the most common toxicity prompting discontinuation.^20,21^ Unfortunately, because RT is administered in the immediate postoperative period before AET is initiated, the decision regarding RT omission must be made before AET tolerance and adherence are known. 

Without access to patient-level data on tolerance to AET, physicians and patients face challenges in making a fully informed decision about forgoing RT. To help address this, we initiated the **P**re-**O**perative **W**indow of **E**ndocrine Therapy to Inform **R**adiation Therapy Decisions (POWER) trial, a phase II, prospective trial. The POWER trial enrolled women aged 65 years and older with stage I ER+ breast cancer. Participants received 90 days of preoperative ET (pre-ET) to assess their tolerance and inform RT decisions based on individual experiences. The primary aim of this study was to evaluate whether patients’ likelihood of pursuing RT and surgeons’ strength of recommendation for RT changed after 90 days of pre-ET compared with their baseline opinions.

## Methods

The POWER trial (NCT0427801) is a single-arm trial conducted at two academic medical centers: the University of Virginia (UVA), which began enrolling patients in April 2020, and Virginia Commonwealth University (VCU), which opened to enrollment in November 2022. The study closed to accrual in December 2023. Eligible participants were women aged 65 years or older with ER+, progesterone receptor (PR)±, HER2-negative invasive breast cancer, and Eastern Cooperative Oncology Group (ECOG) performance status of 0–2. Additional eligibility criteria included a tumor size of ≤2 cm on imaging, clinically normal axillary lymph nodes, and planned BCS as surgical treatment. Patients with prior ET use or previous ipsilateral breast RT treatment were excluded. The co-primary endpoints were the change in both patient preference and surgeon recommendation for RT following 90 days of pre-ET, compared with baseline.

All participants received 90 days of pre-ET, consisting of either tamoxifen or an aromatase inhibitor (AI), as determined by the treating medical oncologist. The specific medication could not be changed during the pre-ET period but could be switched in the adjuvant setting. Patients were encouraged to complete the full 90 days of pre-ET. Those who chose to stop early remained in the study and proceeded directly to BCS. All others underwent BCS following the prescribed pre-ET course. The study did not mandate the inclusion or omission of sentinel lymph node biopsy (SLNB). Following BCS, the decision to proceed with or omit RT was made collaboratively by the treating physicians and the patient. While AET is recommended for all participants in accordance with the standard of care, it was not required by the study protocol.

**Fig. 1:**
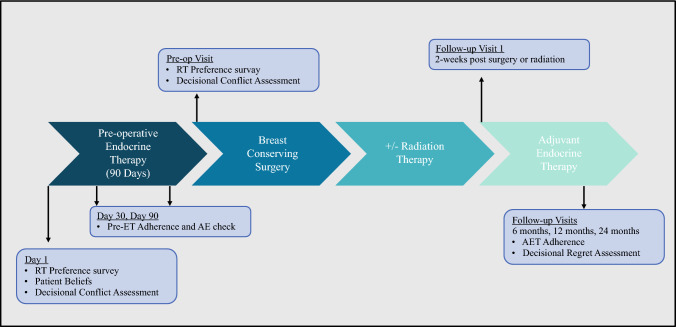
POWER trial schematic. Patients were seen on days 1, 30, and 90 during the pre-ET period. During the adjuvant period, patients were seen 2 weeks after BCS or RT (whichever occurred later) and followed for 24 months. The primary endpoint was evaluated prior to starting pre-ET and at the preoperative visit immediately before surgery. Patients were followed for 24 months postoperatively. On Day 1, patient beliefs were captured using the BIPQ and the UVA Breast Cancer Belief Survey. The Decisional Conflict Scale was completed at Day 1 of pre-ET and at the preoperative visit. The Decisional Regret assessment was completed during the adjuvant follow-up period at the 6-, 12-, and 24-month visits. *pre-ET* preoperative endocrine therapy, *BCS* breast-conserving surgery, *RT* radiation therapy, *BIPQ* Brief Illness Perception Questionnaire, *AE* adverse event

Preoperative study visits occurred on days 1, 30, and 90 during the pre-ET period. Adherence to pre-ET, initiation of AET, and adherence to AET were patient-reported and recorded at each study visit. In the follow-up period, interruptions in AET for any reason (e.g., surgery, drug holidays) were encouraged to be <12 weeks per year. Symptoms experienced with pre-ET were tracked as adverse events (AEs) by the National Cancer Institute Common Terminology Criteria for Adverse Events (version 5). The patients’ preferences and strength of surgeons’ recommendations for RT were assessed using a 4-point Likert scale (1 = weak preference for RT and 4 = strong preference for RT) on Day 1 of pre-ET and at a preoperative visit after completing 90 days of pre-ET. All patients were followed for 24 months after BCS. Follow-up visits were scheduled approximately 2 weeks after either BCS or completion of RT (whichever occurred later), and then at 6, 12, and 24 months after this initial follow-up visit. Participants were considered lost to follow-up if they missed three scheduled visits and could not be contacted by the study site staff (Fig. 1).

For this analysis, participants were assigned to treatment groups based on their AET adherence status at the time of individual follow-up visit. For example, a participant who underwent BCS and RT and initiated AET would initially be categorized in the BCS+RT+AET group; however, if they discontinued AET prior to the 6-month follow-up visit, they would then be reclassified into the BCS+RT group at that time point. This dynamic group approach allowed us to track global trends in the proportion of participants falling within treatment groups throughout the follow-up period.

### Patient-Reported Outcome Questionnaires

Patient-reported outcomes (PROs) were collected using surveys administered at pre-ET visits and follow-up visits. Before starting pre-ET, patients completed a novel UVA Breast Cancer Belief Survey, which included two questions assessing baseline worry about breast cancer and two questions assessing concern about medication adverse effects. All responses were recorded using a 4-point Likert scale: ‘strongly disagree’, ‘disagree’, ‘agree’, and ‘strongly agree’. Participants also completed the Brief Illness Perception Questionnaire (BIP-Q) before taking pre-ET. The BIPQ is a validated 8-question survey (each question scored 0–10, with a maximum total score of 80) that evaluates patients' perceptions of breast cancer.^22^ A score of <42 indicates a perceived low breast cancer threat, 42–49 indicates a moderate threat, and >49 indicates a high threat.

Patients’ decisional conflict and regret were assessed using the validated Decisional Conflict Scale (DCS)^23^ and Decisional Regret Scale (DRS).^24^ DCS is a 16-item survey that measures patient perceptions of uncertainty surrounding treatment choices. Questions are assessed on a 0–4 Likert scale. The total scores are converted to a scale of 0 (no conflict) to 100 (highest conflict). Scores of <25 are associated with low levels of decisional conflict and the ability to make decisions easily. In the POWER trial, decisional conflict was evaluated before and after pre-ET. DRS is a validated 5-item survey assessed on a 0–5 Likert scale that measures self-reported regret after making healthcare decisions. The total DRS score is calculated by subtracting 1 from each item, multiplying the result by 25, and then taking the average of the calculated individual scores. The final score ranges from 0 (no regret) to 100 (high regret). DRS assessments are measured at the 6, 12, and 24 adjuvant follow-up visits. The DCS and DRS evaluations were added to the study protocol in February 2022.

### Statistical Methods

This study was designed to determine whether pre-ET changed either the participant's likelihood of pursuing RT or the surgeon’s strength of recommendation for RT. For the analysis, preference change was made into a binary variable (yes/no); ‘yes’ if the preference for RT differed between the pre-ET and post pre-ET periods in either direction, or ‘no’ if the RT preference did not change. Target accrual of at least 70 evaluable participants provided 90% power to test for a change in preference from a null difference of 5% to an alternative of at least 15%, with a target one-sided 5% level binomial test (by exact numeration) for each group (participant or surgeon) or an overall one-sided 10% type I error rate. No other statistical testing was pre-planned. Secondary outcomes were described using summary measures and graphical displays. In general, descriptive statistics were used to provide a summary of the patient characteristics, patient adjuvant treatment decisions, AEs, decisional conflict and regret, and beliefs and perceptions about breast cancer. Kappa statistics were used to describe general agreement between participants and surgeons, where answers were equated by the strength of the surgeon's recommendation and the patient's likelihood to pursue RT (weak = unlikely; strong = likely). Sankey plots were used to display the change in preference visually. Side-by-side bar plots and clustered bar plots by adjuvant treatment groups were used to visually summarize beliefs and perceptions about breast cancer. All results were produced using SAS 9.4 (SAS Institute, Inc., Cary, NC, USA).

## Results

### Patient and Tumor Characteristics

Eighty-four patients signed informed consent to participate in the trial. One patient was found to be ineligible after signing consent and four withdrew consent and therefore had no follow-up data. Four additional patients were deemed unevaluable due to failure to complete the primary endpoint survey, leaving 75 patients in the primary analysis. Eighty-one percent of these patients were enrolled at UVA. Most participants were White (90.7%), with a mean age of 73.8 ± 6 years. The majority of patient tumors (77.3%) were invasive ductal carcinoma. Nineteen patients had an SLNB at the time of BCS and none were found to have nodal metastases. Most patients took an AI for pre-ET (85.3%) [Table 1].

**Table 1:** Patient and tumor characteristics

Patient or tumor characteristic	*N* (%)/mean (range, IQR, SD)
*Race*	
Black	7 (9.3%)
White	68 (90.7%)
*Ethnicity*	
Hispanic or Latino	1 (1.3%)
Non-Hispanic	74 (98.7%)
Age, years	73.8 (65–92, 8.0, 6.0)
BMI	30.4 (7.0)
*ECOG status*	
0	52 (69.3%)
1	20 (26.7)
2	3 (4.0%)
*Clinical tumor size pre-surgery*	
T1a (0.1–0.5 cm)	9 (12.0%)
T1b (>0.5–1.0 cm)	38 (50.7%)
T1c (>1.0–2.0 cm)	28 (37.3%)
*Pathology tumor size, cm*	
<0.1	6 (8.0%)
0.1–0.5	7 (9.3%)
0.5–1	23 (30.7%)
1.0–2.0	32 (42.7%)
2.0–3.0	6 (8.0%)
>3^a^	1 (1.3%)
*Histologic type*	
IDC	58 (77.3%)
ILC	11 (14.7%)
Mucinous	1 (1.3%)
Other	5 (6.7%)
Progesterone receptor positive	66 (88.0%)
*Lymph nodes*	
N0	19 (25.3%)
Nx	56 (74.7%)
*Histologic grade*	
1	38 (50.7%)
2	36 (48.0%)
3	1 (1.3%)
*Hormonal therapy*	
Anastrozole	16 (21.3%)
Letrozole	48 (64.0%)
Tamoxifen	11 (14.7%)

*BMI* body mass index, *ECOG* Eastern Cooperative Oncology Group, *IDC* invasive ductal carcinoma, *ILC* invasive lobular carcinoma, *IQR* interquartile range, *SD* standard deviation

^a^Invasive lobular carcinoma

### Primary Endpoint: The Change in Patient and Physician Preference for Radiation Therapy

**Fig. 2:**
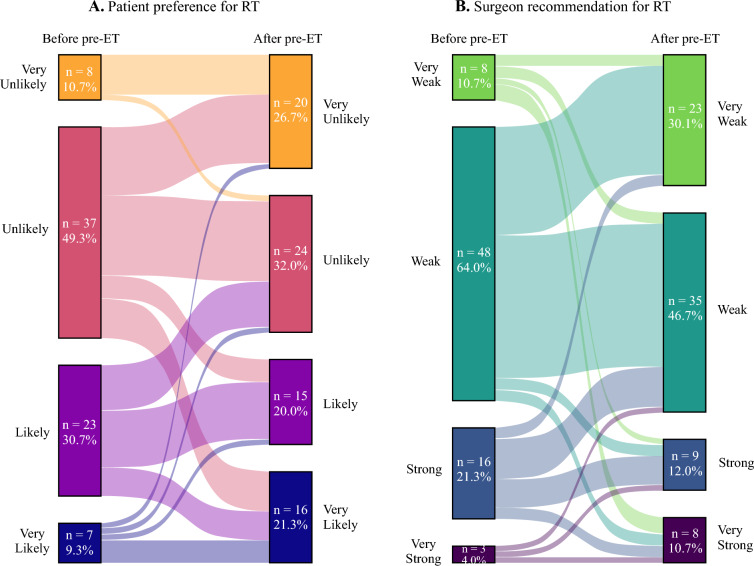
Sankey plot of **a** patient preferences for RT and **b** strength of surgeon recommendation for RT, before and after pre-ET. The size of each ribbon is proportional to the number of patients and surgeons moving from the before pre-ET to the after pre-ET node. The height of each node represents the number of patients or surgeons reporting their particular preference or recommendation for RT before and after pre-ET. *RT* radiation therapy, *pre-ET* preoperative endocrine therapy

Twenty-one participants (28%) changed their preference regarding RT after the pre-ET period. Prior to initiating pre-ET, 60% (*n* = 45) of participants reported they were ‘unlikely’ or ‘extremely unlikely’ to pursue RT. Notably, no patients switched from ‘extremely unlikely’ to ‘likely’ or ‘extremely likely’ to pursue RT. However, 11 of the 37 participants who initially said they were ‘unlikely’ to pursue RT changed their preference to ‘likely’ or ‘extremely likely’ after completing pre-ET. After completing pre-ET, 44 participants (58.7%) reported they were ‘unlikely’ or ‘extremely unlikely’ to pursue RT (Fig. 2).

Following pre-ET, surgeons changed their recommendation for RT in either direction for 18 patients (24%); however, the overall proportion of surgeons who gave a ‘weak’ or ‘extremely weak’ recommendation for RT changed only slightly, from 74.7% before pre-ET to 77.3% after pre-ET (Fig. 2).

The general agreement between patients' preferences and surgeons’ recommendations for adjuvant RT increased after pre-ET. Before pre-ET, 53.3% of participants’ preference for RT matched their surgeon’s recommendation (Kappa = −0.04, 95% confidence interval [CI] −0.25 to 0.18). After pre-ET, concordance of RT preference and recommendation significantly increased to 81.3% (Kappa = 0.59, 95% CI 0.41–0.77).

### Patient Adjuvant Treatment Decisions and Adjuvant Endocrine Therapy Adherence

**Fig. 3:**
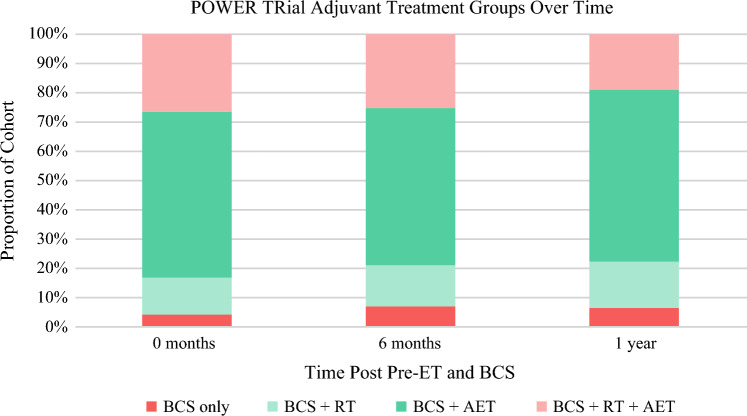
Proportion of participants in each treatment group at three follow-up time points. Time 0 corresponds to the first adjuvant follow-up visit, occurring within 2 weeks of BCS or RT (whichever came later). Participants were grouped by their current adjuvant therapy at the time of the follow-up visit: BCS alone, BCS+RT, BCS+AET, or BCS+RT+AET. Composition of the groups changed over time as some participants discontinued AET. *BCS* breast-conserving surgery, *RT* radiation therapy, *AET* adjuvant endocrine therapy, *pre-ET* preoperative endocrine therapy

Thirty-one (41.3%) participants were treated with RT—45.2% (*n* = 14) with whole-breast irradiation and 54.8% (*n* = 17) with accelerated partial-breast irradiation. Fifty-nine participants (83.1%) initiated AET. Nine (12.7%) participants were treated with RT and did not initiate AET (*BCS+RT*). Nineteen patients (26.8%) underwent RT and initiated AET (*BCS+RT+AET*) following BCS. Forty patients initiated AET and did not have RT (*BCS+AET*). Only 3 (4.2%) participants did not have RT and did not initiate AET (*BCS alone*) [Fig. 3].

Data for AET initiation after BCS and 6-month AET adherence were available for 71 participants. For the present analysis,1-year adherence data were available for 63 patients. Of the 71 participants with 6-month adherence data, 56 (78.9%) were adherent to AET at 6 months, while of the 63 patients with 1-year adherence data, 77.8% were adherent at 1 year. Adherence among the 59 participants who initiated AET after BCS was high, with 94.9% (*n* = 56) still adherent at 6 months and 96.08% (*n* = 49) still adherent at 1 year. Patients who had RT were less likely to be adherent to AET at 1 year than those who did not have RT (54.5% vs. 90.2% adherent at 1 year).

The four patients without primary endpoint data were assessed separately. Three underwent RT and initiated AET (*BCS+RT+AET*) following BCS. One patient underwent RT but did not initiate AET following BCS (BCS+RT).

### Decisional Conflict and Regret

DCS and DRS data were available for 46 and 48 patients, respectively. Overall, study participants were not conflicted regarding their treatment decisions before taking pre-ET, with a mean DCS score of 11.5 ± 12.0. After taking pre-ET, decisional conflict remained low, with a mean DCS score of 13.5 ± 18.2. There were low levels of decisional regret at 6 months (mean 7.8 ± 13.3, *n* = 48) and 1 year postoperatively (mean 5.6 ± 10.2).

### Beliefs and Perceptions about Breast Cancer

**Fig. 4:**
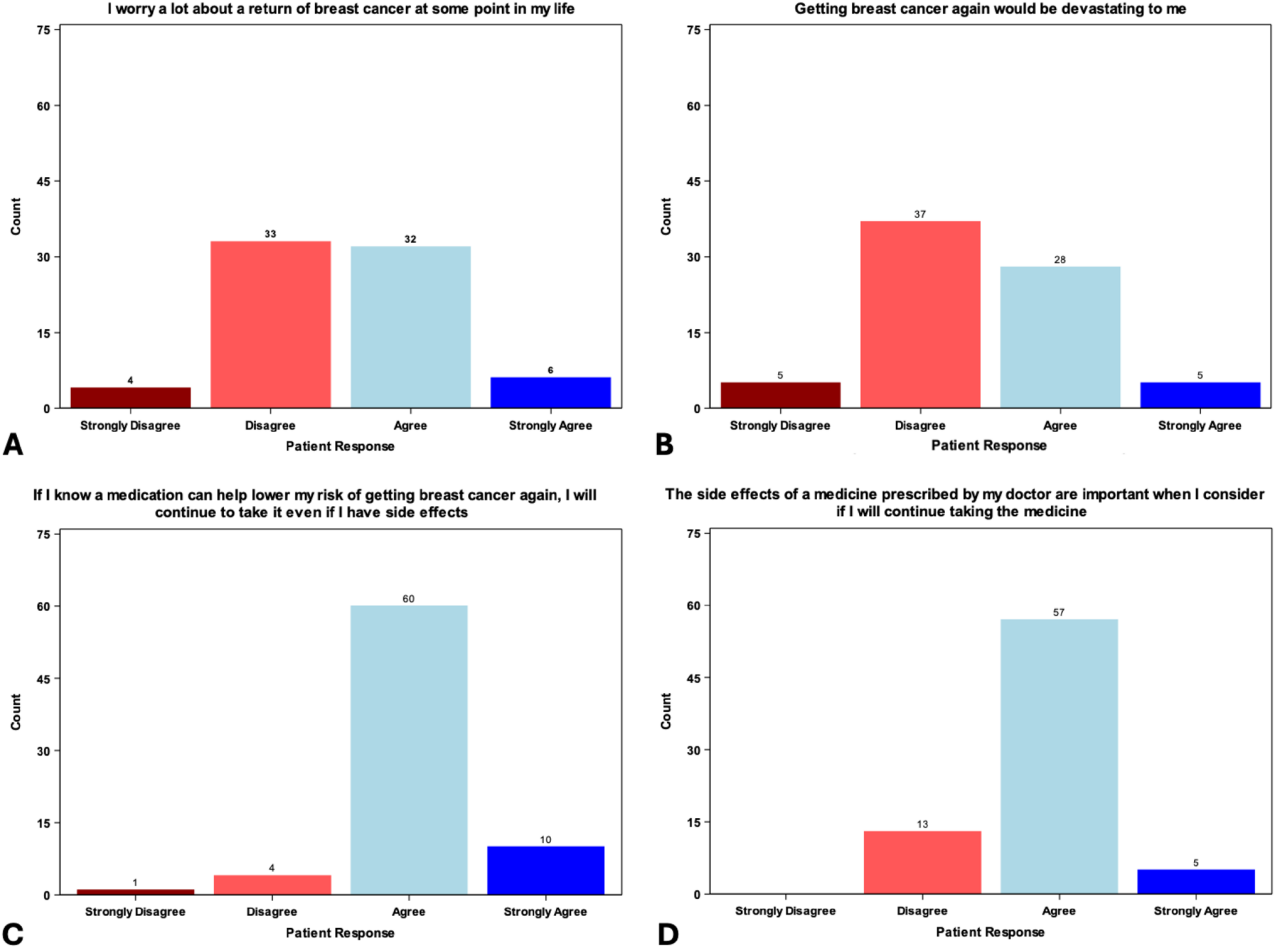
Patient responses to the UVA Breast Cancer Belief Survey, a novel questionnaire assessing baseline worry about breast cancer and concern about medication side effects. Participants were equally divided regarding ‘worry about the return of breast cancer’ **A** and how devastating a breast cancer recurrence would be **B**. Most patients agreed that the side effects of a medication play an important role in their decision to take it **C** and that they would continue to take a medication, despite its side effects, if it would help lower their risk for breast cancer recurrence **D**. *UVA* University of Virginia

Based on the BIPQ, 57.3% of patients perceived breast cancer as a low threat, 28% perceived it as a moderate threat, and 14.6% perceived it as a high threat. The following beliefs were assessed using the UVA Breast Cancer Belief Survey: participants were equally divided regarding ‘worry about the return of breast cancer’ and ‘getting breast cancer again would be devastating’. The majority of patients (82.7%) agreed that the adverse effects of a medication play an important role in their decision to take it. Finally, the majority of patients (93.3%) agreed that they would continue to take a medication, despite its adverse effects, if it would help lower their risk for breast cancer recurrence (Fig. 4).

### Adverse Events

During the 90-day pre-ET period, 35 participants reported treatment-related AEs, with the most common being fatigue, hot flashes, and arthralgias. No grade 4 AEs were observed. Two serious AEs occurred during the pre-ET period—a pulmonary embolism and an acute stroke. Eight patients (10%) discontinued pre-ET early. Of these, six patients stopped due to a non-life-threatening medication adverse effect, one discontinued due to anxiety about delaying surgery, and one stopped due to having an acute stroke while taking tamoxifen (Table 2).

**Table 2:** Adverse events

Adverse event	G1 (%)	G2 (%)	G3 (%)	G4 (%)	G5 (%)	Total (%)
*Ear*						
Vertigo	1 (1)					1 (1)
*Eye*						
Decreased vision		1 (1)				1 (1)
*Gastrointestinal*						
Constipation	2 (3)					2 (3)
Diarrhea	2 (3)	1 (1)				3 (4)
Dry mouth	1 (1)					1 (1)
Oral mucositis	1 (1)					1 (1)
Nausea	4 (5)					4 (5)
*General*						
Limb edema	1 (1)					1 (1)
Fatigue	8 (11)					8 (11)
*Infections*						
Bladder infection		1 (1)				1 (1)
Urinary tract infection	1 (1)					1 (1)
Vaginal infection	1 (1)					1 (1)
*Musculoskeletal*						
Arthralgia	8 (11)	2 (3)				10 (13)
Back pain	1 (1)					1 (1)
Joint effusion	1 (1)					1 (1)
Muscle cramp	1 (1)					1 (1)
Myalgia	1 (1)					1 (1)
*Nervous system*						
Dizziness	1 (1)					1 (1)
Headache	2 (3)	1 (1)				3 (4)
*Psychiatric*						
Agitation	1 (1)					1 (1)
Insomnia	3 (4)		1 (1)			4 (5)
*Reproductive*						
Perineal pain		1 (1)				1 (1)
Uterine pain	1 (1)					1 (1)
Vaginal dryness	2 (3)					2 (3)
Vaginal pain	1 (1)					1 (1)
*Skin*						
Alopecia	7 (9)					7 (9)
*Vascular*						
Arterial thromboembolism			1 (1)			1 (1)
Hot flashes	17 (23)					17 (23)
Hypertension		1 (1)				1 (1)
Thromboembolic event		1 (1)				1 (1)

## Discussion

The POWER trial enrolled women aged 65 years and older with stage I ER+ breast cancer who were deemed appropriate for consideration of RT omission. Participants received 90 days of pre-ET to assess whether this experience influenced patients’ preferences and surgeons’ recommendations for adjuvant RT. Following pre-ET, 28% of patients and 24% of physicians altered their RT preferences, meeting the predetermined threshold for a significant change. Additionally, concordance between patient and physician preferences increased after pre-ET. The pre-ET intervention demonstrated a safety profile consistent with that of previously published neoadjuvant ET trials, with AEs reflective of the known and expected effects of ET.^25–28^ At the 1-year follow-up, treatment patterns were as follows: 6% of patients were treated with BCS alone, 16% were treated with BCS+RT, 59% were treated with BCS+AET, and 19% were treated with BCS+RT+AET. Baseline PROs indicated generally low levels of worry about breast cancer and a willingness to initiate ET despite potential adverse effects. Participants reported low levels of decisional conflict and regret regarding their treatment choices.

Neoadjuvant ET has been shown to effectively downstage disease in postmenopausal women with ER+ breast cancer,^29,30^ supporting its use as a reasonable initial approach in the treatment of early-stage disease in selected patients. While extended durations of neoadjuvant ET are needed to achieve maximal tumor response, the POWER trial utilized a 90-day pre-ET period not for pathologic downstaging but rather to assess patient tolerance. Our previous analysis of PROs during pre-ET revealed that participants experienced adverse effects characteristic of AET, particularly vasomotor and musculoskeletal symptoms.^31^ These findings suggest that the 90-day duration is sufficient to elicit adverse effects and support informed decision making.

Understanding non-adherence to AET remains a challenge as most studies rely on demographic and clinical factors that poorly predict individual behavior.^32^ There is a critical need for real-time, patient-level data on treatment tolerance. The pre-ET period offers a valuable opportunity for both patients and physicians to assess tolerance and make more informed decisions regarding adjuvant therapy. In the POWER trial, concordance between patient and surgeon RT preferences significantly increased following pre-ET. While most patients tolerated pre-ET well and chose RT, a notable subset (24.4%) shifted from a low to a strong preference for RT. This likely reflects a realization that they did not tolerate pre-ET well and may face challenges with long-term adherence to AET, leading them to opt for RT as a critical component to avoid undertreatment and reduce the risk of recurrence.

While the POWER trial aimed to assess how pre-ET influences RT preference, it also impacted actual adjuvant treatment choices and patterns. Analysis of Surveillance, Epidemiology, and End Results (SEER)-Medicare data has shown that only 21% of eligible patients omit RT.^11^ In contrast, 59% of participants in the POWER trial omitted RT, suggesting that pre-ET may facilitate shared decision making regarding RT omission. Notably, while RT omission rates are higher in the POWER trial than in historical data, undertreatment with BCS alone was lower in the POWER trial (*n* = 5, 7% at 6 months) compared with a published rate of 13.8%.^33^ This low rate of undertreatment in the POWER trial is likely due to two factors. First, patients who had poor tolerance to pre-ET often chose to have RT, recognizing their higher likelihood of not initiating or adhering to AET. Second, those who tolerated pre-ET well were more confident in omitting RT and were more likely to remain adherent to AET, reducing the risk of undertreatment. A systematic review reported that 15–20% of tamoxifen users and 5–25% of AI users discontinue therapy within the first year of therapy.^34,35^ In the POWER trial, 94% and 96% of patients who initiated AET remained adherent at 6 months and 1 year, respectively.

Previous studies assessing treatment trends in older women with early-stage ER+ breast cancer, including a retrospective cohort of patients treated at UVA from 2012 to 2022, showed that 42–55% of patients were treated with BCS+RT+AET.^18,33,36–38^ At the 1-year follow-up, only 19% of POWER participants were treated with BCS+RT+AET. For this population, adding both RT and AET increases cost and toxicity without improvement in overall survival,^6,7^ and may therefore represent overtreatment. The phase III randomized EUROPA trial^39^ further supports a shift in practice away from overtreatment. In that study, women aged 70 years and older with stage I, ER+/PR+/HER2− breast cancer undergoing BCS were randomized to treatment with AET or RT. AET was associated with a greater decline in health-related quality of life (HRQOL) than RT. Oncologic outcome data are pending. The design and intent of the EUROPA trial underscore a growing movement toward individualized treatment strategies that minimize overtreatment and prioritize HRQoL. Similarly, the POWER trial results demonstrate that pre-ET exposure supports a more personalized approach, with a greater proportion of patients receiving appropriate treatment and fewer patients being under- or overtreated.

Participants in the POWER trial reported low baseline levels of worry about breast cancer recurrence and a general willingness to take breast cancer-specific medications despite potential adverse effects; however, findings from this trial, along with other clinical trials and real-world studies, indicate that a substantial proportion of patients do not adhere to AET.^15,16,40^ This highlights that initial attitudes, as captured through baseline PROs, do not reliably predict long-term adherence or treatment decisions. The Ottawa Decision Support Framework (ODSF)^41,42^ provides a framework for addressing decisional needs of patients facing complex health decisions using decision support interventions (DESIs). Key DESI evaluation measures include decisional conflict and decisional regret.^42,43^ Decisional conflict is the “simultaneous opposing tendencies within the individual to accept or reject a given course of action”.^41^ Decision regret is a negative emotion associated with regret of healthcare decisions associated with undesired outcomes.^24^ POWER trial participants reported consistently low levels of decisional conflict before and after pre-ET, as well as low levels of decision regret in the adjuvant period. These findings suggest that pre-ET serves well as a DESI, helping patients clarify preferences and make informed choices without leading to a high level of conflict or regret.

This study has some limitations. While there are many reasons for the continued use of RT in this patient population, an overarching concern remains AET adherence. The POWER trial was designed to mitigate this factor. The POWER trial was conducted at two academic institutions located in Virginia, which may not reflect broader practice patterns. Additionally, the study cohort was relatively homogeneous. Most participants identified as White, which limits the generalizability of the results, particularly given documented racial disparities in AET adherence.^44^ Opening the study at a second site (VCU) helped improve participant diversity. Eighteen percent of participants at VCU were Black, compared with 6% at UVA; however, the overall study population remained predominantly White. Regional factors influence patient beliefs, treatment patterns, and preferences, and the study's geographic restriction may limit its applicability to other populations.

## Conclusions

Ninety days of pre-ET significantly influenced both patients’ likelihood of pursuing RT and surgeons’ strength of recommendation for RT. Additionally, there was increased agreement between patients’ preferences and surgeons’ recommendations after pre-ET than before pre-ET. These findings validate pre-ET as an innovative method to inform adjuvant therapy decisions and recommendations. The intentional resequencing of treatments enables patients to assess their tolerance to ET before making definitive decisions about adjuvant therapy. As a natural next step, the ongoing POWER II trial (NCT06507618) is a randomized controlled trial designed to determine the impact of pre-ET on over- and undertreatment. The POWER trials have the potential to create a paradigm shift in the treatment of older women with early-stage ER+ breast cancer. 
